# *Drosophila* XBP1 Expression Reporter Marks Cells under Endoplasmic Reticulum Stress and with High Protein Secretory Load

**DOI:** 10.1371/journal.pone.0075774

**Published:** 2013-09-30

**Authors:** Hyung Don Ryoo, Josepher Li, Min-Ji Kang

**Affiliations:** 1 Department of Cell Biology, New York University School of Medicine, New York, New York, United States of America; 2 Department of Biomedical Science, University of Ulsan College of Medicine, Seoul, Korea; University of Massachusetts Medical School, United States of America

## Abstract

Expression of genes in the endoplasmic reticulum (ER) beyond its protein folding capacity activates signaling pathways that are collectively referred to as the Unfolded Protein Response (UPR). A major branch of the UPR pathway is mediated by IRE1, an ER-tethered endonuclease. Upon ER stress-induced activation, IRE1 splices the mRNA of XBP1, thereby generating an active isoform of this transcription factor. During normal *Drosophila* development, tissues with high protein secretory load show signs of IRE1/XBP1 activity indicative of inherent ER stress associated with those cell types. Here, we report that the XBP1 promoter activity itself is enhanced in secretory tissues of *Drosophila*, and it can be induced by excessive ER stress. Specifically, we developed a *Drosophila* XBP1 transcription reporter by placing dsRed under the control of the XBP1 intergenic sequence. DsRed expression in these *xbp1_p_>dsRed* transgenic flies showed patterns similar to that of *xbp1* transcript distribution. In healthy developing flies, the reporter expression was highest in salivary glands and the intestine. In the adult, the male reproductive organs showed high levels of dsRed. These tissues are known to have high protein secretory load. Consistently, the *xbp1_p_>dsRed* reporter was induced by excessive ER stress caused by mutant Rhodopsin-1 overexpression. These results suggest that secretory cells suffer from inherent ER stress, and the *xbp1_p_>dsRed* flies provide a useful tool in studying the function and homeostasis of those cells.

## Introduction

Eukaryotic cells express most of their membrane and secretory proteins in the endoplasmic reticulum (ER), where those proteins undergo folding before being trafficked to their ultimate destination. Expression of mutant or wild type proteins that exceed the protein folding capacity of this organelle can impose stress that disrupts many aspects of cellular function. In response, most eukaryotic cells are equipped with signaling pathways that can induce the expression of ER quality control genes, which are widely referred to as the Unfolded Protein Response (UPR) [1].

Excessive ER stress and defective UPR underlies a wide variety of diseases in humans [2], [3]. Similarly, a number of human disease models of *Drosophila*, including Retinitis Pigmentosa, Alzheimer’s disease and Amyotrophic Lateral Sclerosis are also associated with ER stress [4], [5], [6], [7], [8]. A UPR pathway that is conserved between yeast, *C. elegans*, *Drosophila* and mammals is mediated by IRE1 and XBP1. IRE1 is an ER-tethered protein with a luminal domain that can bind to misfolded peptides [9]. Upon recognizing misfolded proteins, IRE1 forms oligomers on the ER membrane and activates its cytoplasmic RNAse domain [10], [11]. A major target of IRE1 is the mRNA of the transcription factor *xbp1* [12], [13], [14], [15]. IRE1 mediated cleavage, and a subsequent ligation reaction results in an unconventional mRNA splicing event that occurs in the cytoplasm, and results in the shift of the XBP1 reading frame, thereby generating an isoform of XBP1 that acts as an active transcription factor. Once activated, XBP1 induces the expression of a number of ER quality control genes, including chaperones that fold misfolded proteins, and those involved in the degradation of misfolded ER proteins [16], [17].

We had previously shown that *xbp1* is an essential gene for *Drosophila* development [4]. That observation raised the possibility that there are physiological levels of ER stress associated with normal development. Supporting that idea, we recently reported an XBP1 mRNA splicing reporter that indicated signs of IRE1 activity in healthy tissues of *Drosophila* [18]. Specifically, those tissues with constitutive IRE1 activity included the salivary gland, intestine and the male reproductive system, which are known for their high protein secretory load. Here, we report the development of an *xbp1* transcription reporter, which drives dsRed under the control of the *xbp1* promoter. We find that this reporter is also expressed in cells with high protein secretory load and is further induced by experimentally imposed ER stress. These results suggest that secretory cells suffer from inherent ER stress, and cells respond by inducing the transcription of *xbp1,* in addition to the IRE1-mediated splicing of these mRNAs. These tools may further facilitate the study of secretory protein homeostasis and stress response in *Drosophila*.

## Materials and Methods

### Constructs

The 0.85 kb intergenic sequence between *xbp1* and CG9406 was amplified through genomic PCR. Specifically, the amplified DNA began from the end of CG9406, up to the start codon of the *xbp1* coding sequence. This DNA was subcloned into the pPelican plasmid with a dsRed reporter [19], which we refer to as *xbp1_p_>dsRed*.

### Fly genetics

This study did not involve vertebrate animal models. The *ire1^f02170^* obtained from the Bloomington Stock Center had background lethal mutations, which were cleaned up by outcrossing to the Ryoo lab *w^1118^*. To generate mosaic clones, *ire1^f02170^* was recombined to the *FRT82* chromosome. Maternal zygotic *ire1^f02170^ −/−* flies were generated by utilizing the *ovo^D1^* based protocol [20]. Specifically, we crossed *FRT82, ire1^f02170^* flies to *hs-flp; FRT82, ovo^D1^* flies to generate *ire1 −/−* clones in the germ line of the progeny. The female progeny of this cross were crossed to *ire1^f02170^, xbp1_p_>dsRed/TM6B/TM6B-GFP* males. The maternal zygotic mutants were distinguished by the presence of *xbp1_p_>dsRed* and the absence of GFP. IRE1 activity was assessed by crossing the *UAS-xbp1-EGFP HG* line [18] to the *tubulin>Gal4* driver.

### Immunohistochemistry

anti-Rhodopsin-1 antibody (4C5) was from DSHB of Univ. of Iowa and was used at 1∶500. *xbp1_p_>dsRed* was visualized through natural red fluorescence. For validation purposes, we used anti-dsRed antibody from Clonetech. Rabbit anti-GFP was from Invitrogen.

## Results

### The XBP1 expression reporter is active in highly secretory tissues

**Figure 1:**
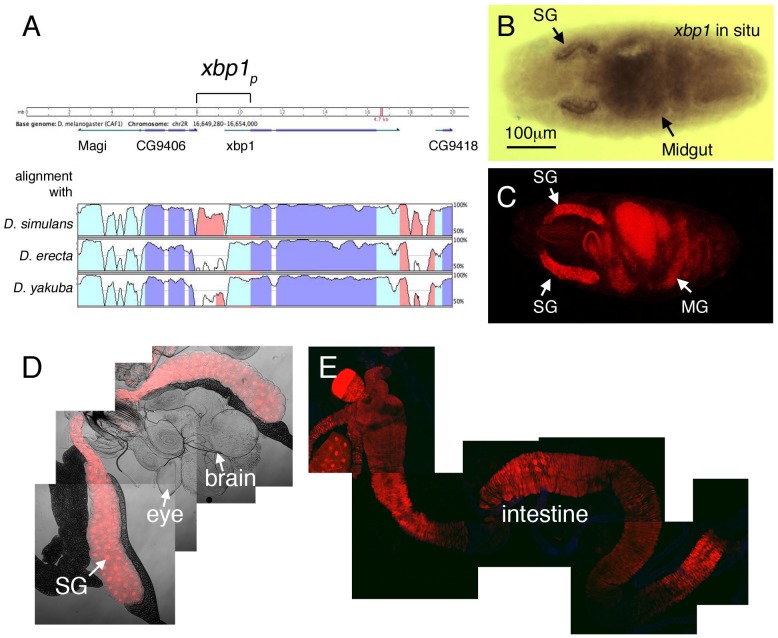
XBP1 regulatory sequence drives dsRed expression in developing secretory tissues. (A) Comparison of the *xbp1* locus sequence between *D. melanogaster, D. simulans, D. yakuba and D. erecta.* Sequence conservation is not only found in the coding sequence of genes (purple) and UTRs (light blue), but also in the intergenic region (red) that may encode enhancers and promoters. The bracket (*xbp1p*) indicates the intergenic sequence that was placed upstream of the dsRed reporter. (B) *xbp1* in situ hybridization in embryos. (C-E) *xbp1_p_>dsRed* reporter expression in the embryo (C), the larval salivary gland (D) and the larval intestine (E). Images in (D, E) are composites of several frames taken with the 10x confocal microscope lens. There was no detectable *xbp1_p_>dsRed* expression in the larval brain and imaginal discs (D). Abbreviations: Salivary Gland (SG), Mid Gut (MG).

In a previous study, we had shown that *xbp1* is primarily expressed in tissues that are notable for their secretory function, including the embryonic salivary gland and the midgut [4] (also Figure 1). To better understand how this gene expression is controlled, we developed an *xbp1* gene expression reporter. Sequence comparison of the *xbp1* locus in a number of *Drosophila* species showed that the intergenic sequence between *xbp1* and its adjacent gene, CG9406, is conserved, raising the likelihood that this region may encode essential regulatory DNA sequences (Figure 1A). Based on this, we placed a dsRed reporter under the control of the *xbp1* upstream intergenic DNA. We found that the transgenic flies expressed dsRed in a pattern that was indistinguishable from that of *xbp1* in situ hybridization (Figure 1B). In embryos, the salivary gland and the midgut showed prominent expression of the *xbp1_p_>dsRed* reporter (Figure 1C). Also in the larva, dsRed was prominently expressed in the salivary gland and the larval intestine (Figure 1D, E).

### IRE1 activity is detected in and beyond the cells expressing the XBP1 transcription reporter

**Figure 2:**
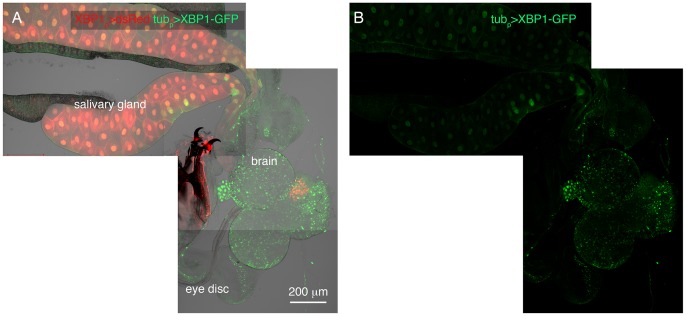
Co-labeling of *xbp1_p_>dsRed* with an IRE1-mediated XBP1 splicing reporter. Shown is a composite image of multiple 10x confocal microscopy images of the 3^rd^ instar larval brain, eye discs and salivary glands. Labeled in red is the *xbp1_p_>dsRed* reporter, and in green is the IRE1-mediated XBP1 mRNA splicing reporter. The latter (IRE1 reporter) was driven ubiquitously through the tubulin-Gal4 driver. (A) Double labeling of dsRed and GFP shows overlap in the salivary glands. On the other hand, the brain and the eye discs primarily show IRE1 activity without XBP1 expression. (B) A GFP only channel of the image shown in (A).

In a previous study, we had described another reporter suitable for detecting IRE1’s enzymatic activity. That reporter took advantage of the fact that IRE1 mediated XBP1 mRNA splicing results in a frame shift in translation. By fusing GFP C-terminal to the XBP1 coding sequence, we were able to have GFP express in frame only in cells that have active IRE1 [18]. When this splicing sensor was expressed ubiquitously through the *tubulin-Gal4* driver, we were able to detect IRE1 activity in the secretory cells that express *xbp1_p_>dsRed* (Figure 2A) [18]. These observations suggest that *xbp1_p_>dsRed* marks many cell types with inherent IRE1/XBP1 pathway activity.

Intriguingly, we noticed that many cells in the brain show signs of IRE1 activity, but do not show *xbp1_p_>dsRed* expression (Figure 2). That observation suggests that there are cells with active IRE1, but not its downstream target XBP1.

### The XBP1 reporter is highly expressed in the adult male reproductory organs

**Figure 3:**
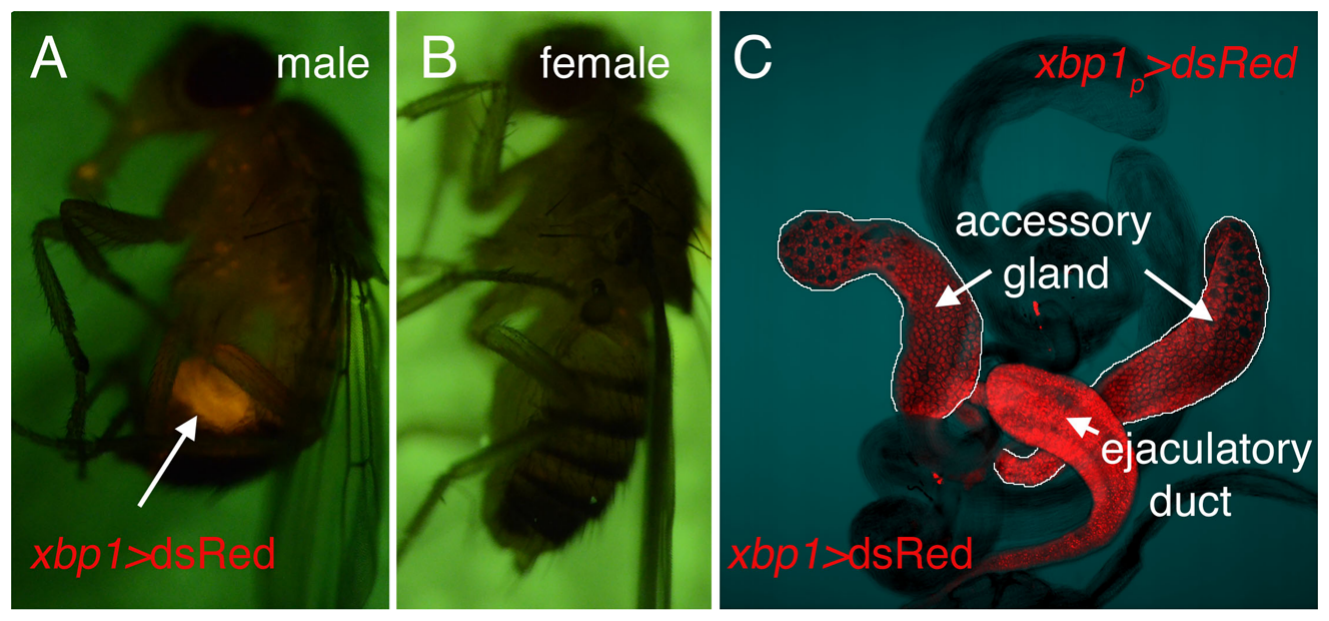
*xbp1_p_>dsRed* expression in the male reproductive system. Prominent dsRed signal was found in the adult abdomen of males (A), but not in females (B). Dissected adult tissues show the strongest dsRed signal in the accessory gland and the ejaculatory duct (C).

In adults, the *xbp1_p_>dsRed* reporter was expressed at high levels in the male abdomen, but not in females (Figure 3A, B). Upon dissection, we found prominent expression of the *xbp1_p_>dsRed* in the male reproductory organs within the abdomen. Specifically, dsRed fluorescence was found in the accessory glands and the ejaculatory duct, but not in the testis itself. Notably, the accessory gland and the ejaculatory duct are known for their high secretory activity (see Discussion).

### The XBP1 promoter is activated by excessive ER stress

**Figure 4:**
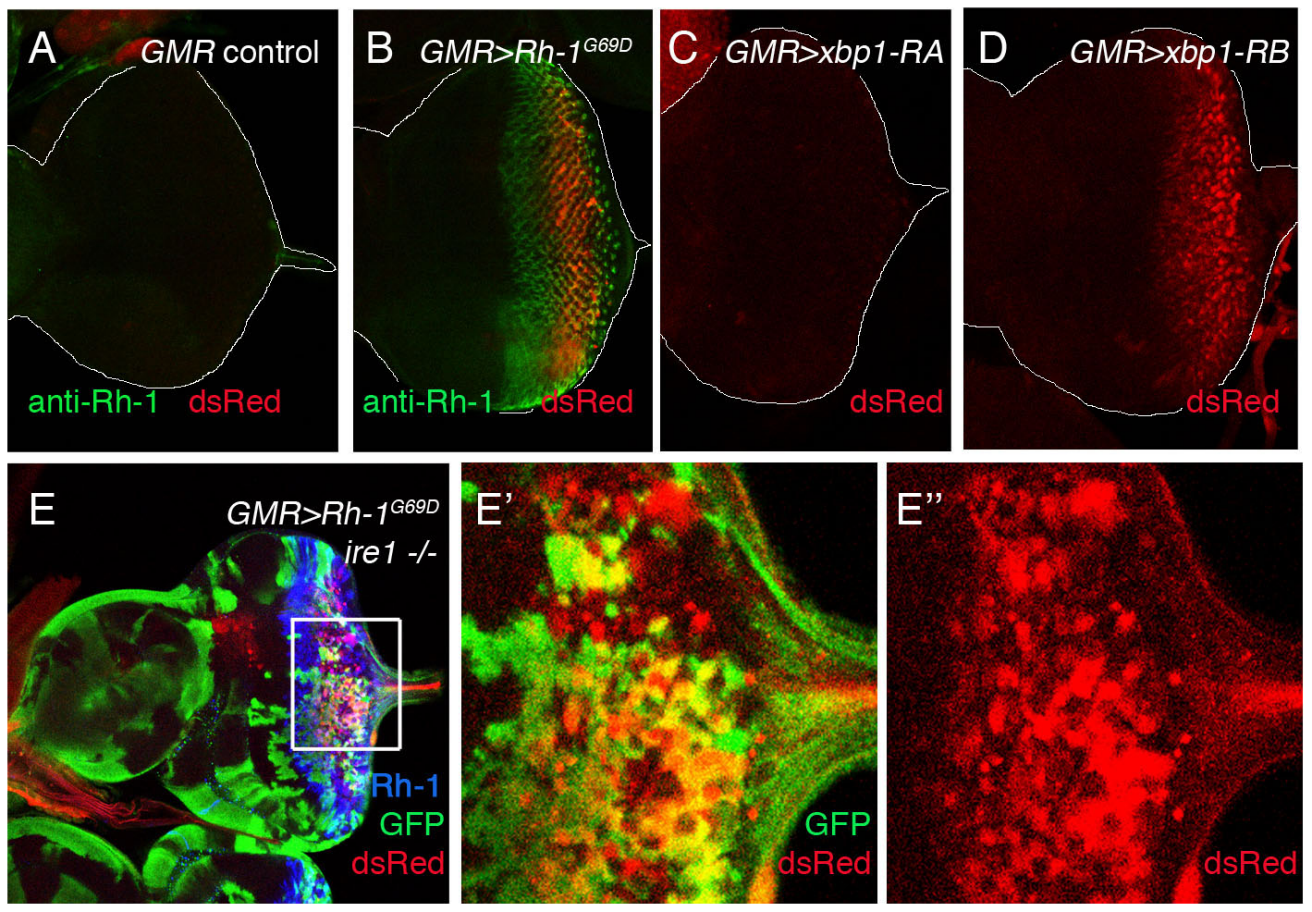
*xbp1_p_>dsRed* expression is induced by misfolded membrane proteins. Shown are larval eye imaginal discs with *xbp1_p_>dsRed* (red). Control eye discs show no basal level of reporter activity (A). *xbp1_p_>dsRed* is induced by the overexpression of a mutant membrane protein, Rhodopsin-1^G69D^ (green) (B), or the spliced *xbp1* isoform *xbp1-RB* (D) in the eye using the *GMR* promoter. On the other hand, the unspliced *xbp1* isoform, *xbp1-RA*, did not induce *xbp1_p_>dsRed* expression (C). (E) *ire1* is not required for *xbp1_p_>dsRed* induction by mutant Rhodopsin-1^G69D^. *ire1* mosaic clones were generated in the eye discs and marked by the absence of GFP. (E’, E’’) show higher magnification images of the inset in (E). *GMR* promoter driven Rhodopsin-1^G69D^ (blue) induces *xbp1_p_>dsRed* in both *ire1+* and *ire1 −/−* cells, although the intensity of dsRed is moderately reduced in *ire1 −/−* clones. (E’’) is a dsRed only channel of (E’).

In a previous study, we had shown that the IRE1/XBP1 pathway can induce the ER chaperone, BiP, in imaginal discs [4]. However, we found no detectable expression of *xbp1_p_>dsRed* in that tissue (Figure 1D). Such inconsistencies led us to explore the possibility that *xbp1_p_>dsRed* is induced in these tissues primarily in response to ER stress. To impose ER stress, we expressed in larval eye discs a mutant membrane protein, Rhodopsin-1^G69D^, which fails to fold properly in the ER [4], [21]. Under these conditions, we found *xbp1_p_>dsRed* induction in that tissue (Figure 4A, B), supporting the idea that *xbp1* promoter itself is induced in response to ER stress.

Interestingly, we found that the IRE1/XBP1 pathway can induce *xbp1_p_>dsRed*. Specifically, we activated the IRE1/XBP1 pathway by overexpressing the spliced isoform of XBP1 (*xbp1-RB*)[22] in *Drosophila* eye discs, which led to the induction of *xbp1_p_>dsRed* (Figure 4C, D). Conversely, we inactivated the IRE1/XBP1 pathway by generating mosaic clones of *ire1^f02170^* mutants. There is a single *ire1* gene in *Drosophila*, unlike the presence of two IRE1 genes in humans. *Drosophila ire1^f02170^* is a null allele with a PiggyBac transposon inserted within the coding sequence [23]. When mutant Rhodopsin-1^G69D^ was expressed in the eye discs with *ire1^f02170^ −/−* mosaic clones, we still observed *xbp1_p_>dsRed* induction, albeit at a reduced level (Figure 4E-E’’). These results suggest that *xbp1_p_>dsRed* expression is controlled by the UPR, but there are pathways other than IRE1/XBP1 that play redundant roles in this process.

### The XBP1 expression reporter is expressed in the *ire1 −/−* larva

**Figure 5:**
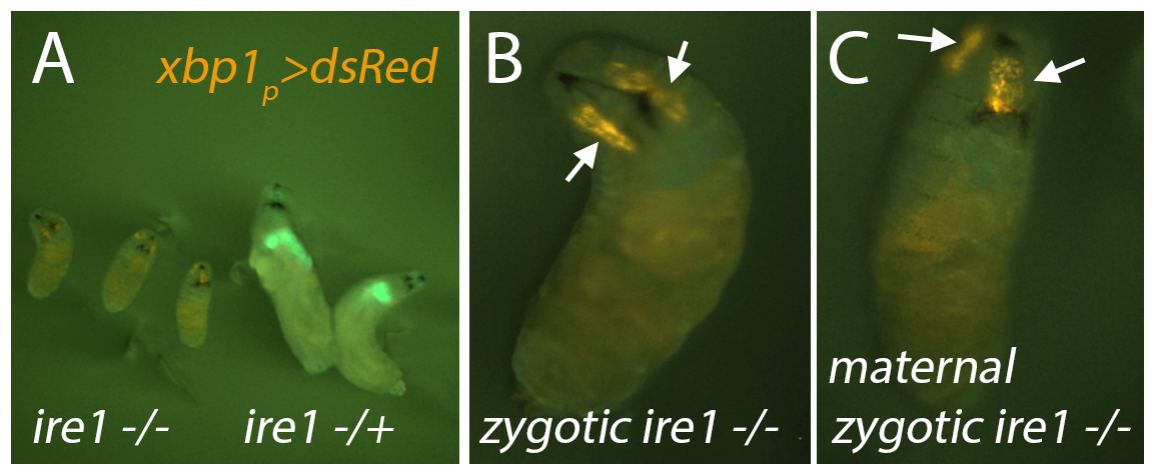
*xbp1_p_>dsRed* is expressed in the salivary gland of *ire1 −/−* larva. (A) Shown are 48 – 72 hour larvae of the *ire1^f02170^ −/−* (left three) and *ire1 −/+* (right two) genotypes. *xbp1_p_>dsRed* was recombined to the *ire1^f02170^* chromosome to facilitate genotyping. The *ire1+* chromosome is marked with GFP. No *ire1 −/−* larvae were found to grow beyond this 1^st^ instar larval stage. (B, C) A higher magnification view of an *ire1 −/−* larva. *xbp1_p_>dsRed* expression in the salivary gland is visible (arrows) in the *ire1 −/−* zygotic (B), as well as maternal, zygotic mutants (C).

We further examined the role of *ire1* in *xbp1_p_>dsRed* expression of developing larvae. At 48 – 72 hours after egg laying, the *ire1^f02170^ −/−* larvae were significantly smaller than that of the *ire1 −/+* siblings, as the homozygous mutant flies failed to develop beyond the 1^st^ instar larval stage (Figure 5). Live *ire1^f02170^ −/−* larvae were no longer found after 72 hours of egg laying.

As XBP1 is highly expressed in salivary glands, we examined whether the salivary glands form at all in the *ire1^f02170^ −/−* flies. To test this, we introduced the *xbp1_p_>dsRed* reporter into the background of *ire1* mutants. The red fluorescence of dsRed was readily detected in these mutants (Figure 5B). To test whether there is a maternal *ire1* mRNA contribution that allows the formation of the salivary gland, we generated maternal zygotic *ire1* mutant flies, which also showed 1^st^ instar larval lethality. These larvae still retained the *xbp1_p_>dsRed* in the salivary gland (Figure 5C), indicating that *ire1* is neither required for the salivary gland formation nor the expression of *xbp1_p_>dsRed* in this tissue.

## Discussion

Here, we report the development of an XBP1 expression reporter, which marked cells and tissues with high protein secretory load. Among the tissues with *xbp1_p_>dsRed* expression are salivary glands that secrete large amounts of digestive enzymes. Intestines of embryos and larvae were also prominent organs with *xbp1_p_>dsRed* expression. The intestine secretes a variety of proteins, not limited to those involved in digestion, but also in anti-microbial activity. In fact, studies in *C. elegans*, mice and humans indicate that *xbp1* plays an essential role in maintaining the integrity of anti-microbial peptide secreting cells, and mutation of *xbp1* in humans enhances risks for the inflammatory bowel syndrome [24], [25]. We also observed high *xbp1_p_>dsRed* expression in the male reproductive organs, such as the accessory gland and the ejaculatory duct. The *Drosophila* accessory gland is a tissue that produces a number of secretory proteins that are referred to as ACPs (Accessory Gland Proteins). There are now more than 50 ACPs that have been identified in *Drosophila*, indicating that the accessory gland is a major secretory tissue [26], [27]. Similarly, the ejaculatory duct also produces secretory proteins, including those that have anti-microbial activity [28]. These observations are consistent with the idea that XBP1 is expressed in cells with high secretory load. In addition, this promoter was induced by excessive ER stress. The latter property may be useful to researchers seeking facile methods to detect ER stress in live *Drosophila* tissues.

The pattern of the *xbp1_p_>dsRed* reporter largely overlapped with the XBP1 mRNA splicing reporter that we described in a recent publication (Figure 2) [18]. The tissues that showed signs of XBP1 mRNA splicing included the larval salivary gland, intestine and the adult accessory glands [18]. Intriguingly, we did not see *xbp1_p_>dsRed* expression in a few cell types that showed the XBP1 mRNA splicing reporter activity. The cell types of the latter class include a number of glial cells in the embryonic and larval brain (compare Figure 1D and [18]). It is noteworthy that the previously described XBP1 mRNA splicing reporter was expressed in all somatic cells using the beta tubulin promoter, even in those tissues with no detectable *xbp1* transcripts. Thus, the splicing reporter did not reflect the expression pattern of *xbp1,* but rather, reported possible IRE1 activity in tissues. Taken together, the difference in the two reporter activities indicate that there are IRE1 active cells (such as certain glial cells) that do not have *xbp1* mRNA. Recent studies indicate that the cytoplasmic RNase domain of IRE1 can cleave mRNAs other than that of *xbp1,* perhaps to reduce the load of new protein synthesis into the stressed ER [29], [30], [31], [32]. Strikingly, certain species like *S. pombe* do not encode an XBP1 ortholog, and IRE1’s main role in that species is to engage in the unconventional activity of degrading mRNAs [33]. Based on this, we speculate that the IRE1 activity in certain glial cells may be engaged in an XBP1-independent role for cellular homeostasis.
